# Development and Validation of In Vitro Assessment Protocol of Novel Intravenous Nanoemulsions for Parenteral Nutrition

**DOI:** 10.3390/pharmaceutics17040493

**Published:** 2025-04-08

**Authors:** Joanna Czerniel, Aleksandra Gostyńska-Stawna, Karina Sommerfeld-Klatta, Tomasz Przybylski, Violetta Krajka-Kuźniak, Maciej Stawny

**Affiliations:** 1 Department of Pharmaceutical Chemistry, Poznan University of Medical Sciences, Rokietnicka 3, 60-806 Poznan, Poland; jczerniel@ump.edu.pl (J.C.); mstawny@ump.edu.pl (M.S.); 2 Department of Toxicology, Poznan University of Medical Sciences, Rokietnicka 3, 60-806 Poznan, Poland; ksommerfeld@ump.edu.pl; 3 Doctoral School, Poznan University of Medical Sciences, Bukowska 70, 60-812 Poznan, Poland; 4 Department of Pharmaceutical Biochemistry, Poznan University of Medical Sciences, Rokietnicka 3, 60-806 Poznan, Poland; vkrajka@ump.edu.pl

**Keywords:** bioethical standards, lipid droplet size, compatibility, hemolysis, MTT

## Abstract

**Background:** Parenteral nutrition (PN) is a lifesaving therapeutic approach for patients unable to meet nutritional needs through oral or enteral routes. Lipid nanoemulsions (NEs), a critical component of PN, provide essential fatty acids and influence the formulation’s physicochemical properties. Advances in drug delivery systems have led to novel intravenous NEs with improved stability, purity, or ability for nutrient/active substance delivery. Due to scattered information and the lack of a standardized protocol for testing intravenous lipid NEs, this study aimed to develop a robust assessment method. The protocol focuses on characterizing the physicochemical properties, stability, and biological efficacy of novel NEs while adhering to bioethical standards. **Methods:** Four NEs were formulated based on fatty acid profile analysis, and to demonstrate the applicability of our protocol, each NE utilized a distinct emulsifier system. A comprehensive in vitro assessment protocol integrating multiple analytical techniques was employed to evaluate their performance. **Results:** The mean droplet diameter (MDD) of developed NEs ranged from 180.7 to 185.9 nm, significantly smaller than commercial formulations (249.6–335.4 nm). PFAT5 remained below 0.02%, except for ILE-HS (0.12%), and the zeta potential (ZP) was below −29.8 mV. The freeze–thaw stability constant (K_F_) of developed NEs was in the range of commercial formulation, and the sterilization stability constant (K_S_) was below 10, except for ILE-HS (23.61 ± 1.65). Injectability tests confirmed that ILE-ELP and ILE-T could be infused at 50 mL/h using an intravenous access with a minimum diameter of 21 G. Hemolytic activity met the strictest criteria (<5%), and MTT assays showed higher cell viability at low concentrations for all NEs except ILE-ELP. **Conclusions:** The developed five-step protocol provides a unified framework for assessing intravenous lipid NEs, allowing for the selection of NEs with the highest potential for further in vivo assessment.

## 1. Introduction

Parenteral nutrition (PN) is a medical intervention that delivers nutrients directly into the bloodstream, bypassing the gastrointestinal tract. This procedure is crucial for patients who cannot receive oral or enteral nutrition and is classified as a lifesaving intervention [1]. Traditionally, PN involves intravenous administration of macronutrients (amino acids, glucose, fatty acids), electrolytes, and other essentials such as vitamins and trace elements in one formulation, namely, PN admixture. Intravenous lipid emulsion is an integral part of such an admixture. Its use allows the maintenance of an energy balance by reducing the carbohydrate load and providing essential fatty acids. Due to the application of intravenous lipid emulsions in the medical field, they must meet appropriate safety standards [1,2,3]. Several intravenous lipid emulsions are available on the market, primarily differing in the composition of their oil phases. However, their long-term administration is associated with potential side effects, including central venous thrombosis, catheter-related bloodstream infections, and intestinal failure-associated liver disease (IFALD). IFALD spectrum refers to a range of hepatobiliary complications, including steatosis, steatohepatitis, fibrosis, cirrhosis, cholestasis, cholelithiasis, and cholecystitis. The genesis of this phenomenon is multifactorial, but the mechanisms are not fully understood. However, it is known that one of the primary contributors to this phenomenon is the use of emulsion based on phytosterol-rich soybean oil [4,5].

Given the above context and ongoing advancements in drug delivery systems, multiple research efforts are underway to develop novel intravenous nanoemulsions (NEs). These aim to offer improved stability or purity, enhanced fatty acid balance, and/or increased capacity for delivering active substances. Introducing a novel intravenous NE for use as a component of PN must be preceded by a series of examinations to meet the requirements for this drug formulation. Despite the long history of lipid emulsion commercialization, there is still a lack of a unified evaluation framework that integrates physicochemical characterization, stability assessment, intravenous compatibility testing, and in vitro toxicity studies into a single protocol. The United States Pharmacopeia (USP) and European Pharmacopoeia (Ph. Eur.) clearly define some requirements [2,6], and other crucial parameters are described in the scientific literature [7]. It is necessary to study the physicochemical properties, such as lipid particle size, pH, and osmolarity, since the administration of intravenous lipid NE with inappropriate physicochemical properties, for instance, the considerable particle size of lipid droplets, can cause capillary embolism (including pulmonary embolism) and a local or systemic inflammatory reaction [8]. It is equally important to verify the feasibility of intravenous administration as a PN component and conduct preliminary stability studies to avoid difficulties in application and complications that would be revealed during in vivo studies. Moreover, the administration of intravenous lipid NEs is associated with a heightened risk of hepatic complications, including steatosis, cholestasis, and even liver failure. Therefore, it is essential to perform in vitro toxicity tests before the administration of such formulations in vivo [3,7,9]. Although the studies mentioned above are separately reported in the literature by different researchers, there is no single clearly delineated path of preliminary studies for developing novel intravenous NEs. Moreover, due to its rich composition, PN is one of the most complex drugs, which generates a high risk of interactions in the pharmaceutical phase. For this reason, conducting compatibility tests between PN and possible new components of this PN admixture seems obligatory. Admittedly, this type of testing is already being introduced in emulsion studies, but there is still no common standard to facilitate the work of researchers [3,10,11]. The preparation and testing of novel NEs are crucial due to the limitations and side effects of currently used intravenous NEs for PN. The creation of a protocol that includes the next steps in the testing of intravenous NEs, which was created based on an analysis of current reports and requirements, can be an invaluable tool to streamline the planning and implementation of work that contributes to the development of research on NEs used in PN.

By implementing rigorous bioethical standards and adhering to the 3R (Replacement, Reduction, and Refinement) principles, which provide recommendations for limiting and optimizing animal testing [12,13], we seek to establish a robust protocol to ensure consistent and reliable safety assessments for NEs used in PN. Our study was designed to define the minimum requirements for NE evaluation before in vivo administration. We evaluated the proposed protocol on NEs designed and prepared by us, potentially intended as a component of the PN admixture. The lipid phase of developed NEs was designed to guarantee the appropriate fatty acid profile and contained four different co-emulsifiers to accurately determine the impact of these variations throughout the tests proposed in the developed assessment protocol.

## 2. Materials and Methods

### 2.1. Materials

Fish oil, medium chain triglycerides (MCTs), Lipoid^®^ E 80, and sodium oleate were kindly gifted by Lipoid^®^ GmbH (Ludwigshafen am Rhein, Germany). Water for injection, Lipidem, Omegaflex^®^ Special without electrolytes (OSE (−)), and Omegaflex^®^ Special with electrolytes (OSE (+)) were purchased from B. Braun Melsungen AG (Melsungen, Germany). Intralipid^®^ and SMOFlipid were purchased from Fresenius Kabi AB (Uppsala, Sweden). Cannabis seed oil was purchased from Molpharma (Ustroń, Poland), rapeseed oil was purchased from Bielmar Sp. z o.o. (Bielsko-Biała, Poland), and sunflower oil was purchased from Eol Polska Sp. z o.o. (Szamotuły, Poland). Kolliphor^®^ HS 15 (KOL-HS) and Kolliphor^®^ ELP (KOL-ELP), Tween^®^ 80 (T80), and α-tocopherol were purchased from Sigma-Aldrich (Schnelldorf, Germany), and glycerol was purchased from POCH S.A. (Gliwice, Poland). All chemicals were analytical or high-performance liquid chromatographic-grade reagents.

### 2.2. Study Design

**Figure 1:**
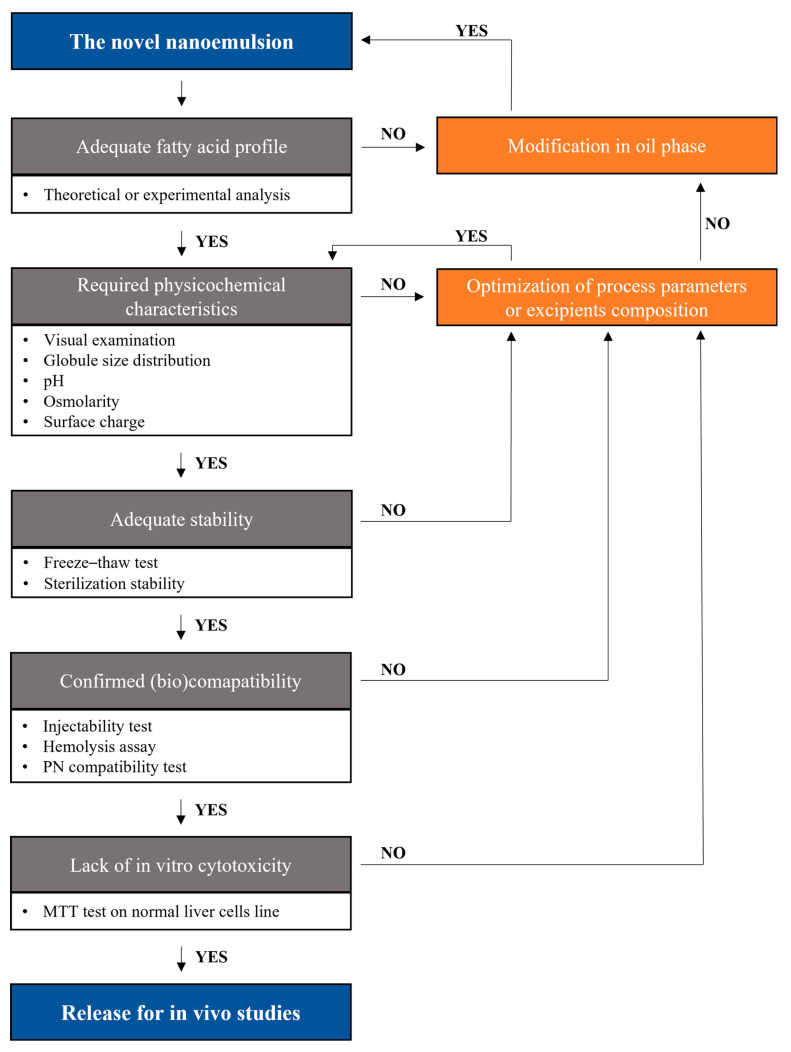
The assessment protocol of novel intravenous (IV) lipid nanoemulsions for parenteral nutrition (PN).

Four novel NEs prepared using different emulsifier systems were tested according to the developed protocol. The oil phase of novel formulations was designed to meet patient needs regarding fatty acids. The chosen oil phase was further utilized to prepare four NEs with different emulsifier systems to study the effect of surfactants on critical physicochemical properties that affect the intravenous administration potential of the developed NEs. The studied NEs were used as a showcase for creating and evaluating an in vitro assessment protocol specifically tailored to novel intravenous NEs intended for PN. The study was divided into five stages: (1) analysis of the fatty acid composition—crucial in planning an experiment to create emulsions with fatty acid profiles that meet the needs of individuals; (2) determining the required physicochemical properties—a preliminary analysis to answer whether the prepared NE meets the basic standards specified for intravenous administration; (3) determining the preliminary stability of the formulation—necessary tests that take little time but can give an initial stability assessment and predictions of NE behavior during storage in long-term stability tests, which can significantly increase the efficiency of ongoing studies; (4) verifying the possibility of intravenous administration as a component of PN—crucial tests that provide a preliminary answer as to whether the formulation has the potential for intravenous administration, which minimizes the amount of error that could arise with in vivo testing; and (5) assessing the toxicity effect in vitro—the last study that should be conducted by introducing animal studies, which gives a preliminary picture of the toxicity and effectiveness of the developed NE for PN. The proposed evaluation protocol is schematically shown in Figure 1. Our proposed assessment protocol, based on an analysis of the scientific literature, is not only designed to optimize the work of researchers but also fits into the 3R principles for in vivo research.

### 2.3. Preparation of the Nanoemulsions

The compositions of the prepared NEs are shown in Table 1. The composition of the aqueous phase was developed by analyzing the composition of commercially available lipid emulsions, while the oil phase was designed from scratch by reviewing the literature on the fatty acid composition of various oils [3,14,15]. The formulation of an optimal ratio of different oils is intended to achieve a balanced lipid profile, ensuring a tailored fatty acid composition that meets the specific medical needs of patients. The oil phase consisted of α-tocopherol, MCT, sunflower, canola, hemp seed, and fish oil. The aqueous phase contained egg yolk lecithin, sodium oleate, glycerol, water, and a selected co-surfactant. Each emulsion was based on egg yolk lecithin (Lipoid^®^ E 80) at a concentration of 1.2% (*w*/*v*) following FDA recommendations and the lecithin content of commercially available intravenous emulsions [3,16]. The first NE (ILE) was based on lecithin alone, and it was the reference for the other three formulations: ILE-HS, ILE-ELP, and ILE-T, which contained 0.25% (*w*/*w*) Kolliphor HS15, Kolliphor ELP, and Tween 80 as co-surfactant, respectively. The non-ionic co-emulsifiers were added to evaluate their effect on the physicochemical properties and the safety of the developed NEs. The rationale behind the choice of their use was based mainly on their high safety profile and FDA registrations for intravenous formulations. Tween 80 (polysorbate 80) is one of the best-known and most widely used non-ionic emulsifiers. The other co-emulsifiers used in the developed ILEs were Kolliphor HS15 and Kolliphor ELP, which belong to the polyethylene glycol (PEG) family of fatty esters. They are FDA-approved for intravenous administration and show a high safety profile and solubilization efficiency [16,17,18,19]. High-pressure homogenization preceded by ultrasonic homogenization was applied to prepare the NEs. Concisely, the components of the aqueous and oil phases were combined separately, covered, and heated to 65 °C while continuously stirring at 600 rpm for 40 min. After dissolution, the aqueous phase was subjected to ultrasonic homogenization (Sonopuls HD 2070, Bandelin electronic GmbH & Co., KG, Berlin, Germany), during which the oil phase was slowly dripped at a 50% amplitude (60 s on, 30 s off) for 9 min to obtain a coarse emulsion. The prepared emulsions were subjected to high-pressure homogenization at 1000 bar for 15 cycles (GEA PandaPLUS 2000, GEA Niro Soavi, Düsseldorf, Germany). During this process, the formulations were cooled in ice. The developed NEs were enclosed in injection vials under a nitrogen atmosphere, followed by thermal sterilization. The sterilization was performed in a steam sterilizer for 20 min at 121 °C. All NEs were stored at 4 ± 1 °C without light exposure.

### 2.4. Physicochemical Characterization of NEs

For physicochemical characterization, parameters such as visual examination, mean droplet diameter (MDD), polydispersity index (PDI), the percentage of fat residing in globules larger than 5 µm (PFAT5), pH, osmolality (OSM), and zeta potential (ZP) were determined and compared with acceptance criteria.

According to the Ph. Eur. [6], all samples underwent visual assessment under diffuse daylight against white and black backgrounds. This observation allowed for the evaluation of transparency and the detection of early signs of destabilization caused by creaming. Subsequently, the size of lipid droplets in the NEs was evaluated following the requirements of the USP as specified in monograph 729 [2]. Two analytical methods were applied for this purpose. Method I was used to determine the MDD and PDI using the Zetasizer Nano ZS (Malvern Instruments, Worcester, UK) based on the dynamic light scattering (DLS) method. The device is equipped with a 633 nm laser with a fixed scattering angle of 173°, and the temperature of the detection chamber was kept constant at 25 °C during the measurements. Method II focused on assessing the large-diameter fraction, expressed as PFAT5, using the light obscuration and single-particle optical sensing method with the PAMAS SVSS particle counter (Partikelmess- und Analysesysteme GmbH, Rutesheim, Germany) equipped with the PAMAS HCB-LD-25/25 volumetric sensor. The PFAT5 values were calculated following the procedure described by Peng et al. [20]. Both methods required sample dilution prior to measurement. For Method I, the samples were diluted at a 1:100 ratio in water for injection, while for Method II, the dilution ratio was 1:2000 in water for injection. The surface charge of lipid droplets in the NEs was determined through ZP analysis using the Zetasizer Nano ZS. The ZP value was calculated using the Smoluchowski equation based on electrophoretic mobility. The pH of the NEs was measured with a SevenCompact pH meter (Mettler Toledo, Columbus, OH, USA), and osmolality was determined using the freezing point method with an Osmometer 800 CLG (Tridentmed, Warsaw, Poland). All measurements were performed in triplicate to ensure reliability.

### 2.5. Preliminary Stability Studies

A freeze–thaw test, involving three cycles of freezing and thawing the formulation, and the sterilization stability test were performed to evaluate the stability. The freeze–thaw test was conducted in the multi-reactor crystallizer Crystal 16 (Avantium Technologies, Amsterdam, The Netherlands). An amount equal to 1 mL of developed NEs was tested at a temperature change rate of 1.0 °C/min. The temperature was varied from −10 to 40 ± 1 °C and maintained at the maximum and minimum levels for 10 min. The effect on NEs stability was determined by measuring MDD, ZP, PDI, OSM, and PFAT5; additionally, based on the MDD values determined in predetermined intervals, the freeze–thaw stability constant (K_F_) was calculated using the following formula:

(1) $$K_{F}=\frac{\sqrt{\frac{1}{n-1}\times \sum_{i=1}^{n}{\left(D_{i}-D_{m}\right)}^{2}}}{D_{m}\times 100}$$

where *n* is the total number of times that the particle size was measured in the freeze–thaw test, *D_i_* is the particle size measured in the test, and *D_m_* is the total mean value of the particle size [21].

In the case of the sterilization stability test, the MDD of the studied NEs was determined before and after the sterilization process, and the sterilization stability constant (K_S_) was calculated as follows:

(2) $$K_{S}=\frac{\sqrt{{\left(D_{b}-D_{m}\right)}^{2\;}+{\left(D_{a}-D_{m}\right)}^{2}}}{D_{m}\times 100}$$

where *D_a_* and *D_b_* are the particle sizes measured before and after sterilization, respectively, and *D_m_* is the average value of *D_b_* and *D_a_* [21].

The mid-term stability study was conducted to validate the results obtained in the preliminary stability test. For this reason, tested NEs were stored for 240 days at 4 ± 1 °C without exposure to light. The corresponding aliquots of NEs for testing at fixed intervals were analyzed for MDD, PDI, ZP, pH, and OSM. All samples were prepared in triplicate, and results were reported as mean ± standard deviation (SD).

### 2.6. Verification of the Possibility of Intravenous Administration as a Component of PN

#### 2.6.1. Injectability

The injectability test was performed following the methodology described by Gostyńska et al. [22]. The test assessed the pressure generated by the emulsions during administration through various needle sizes using a syringe infusion pump (Perfusor Compact Plus, B. Braun Melsungen AG, Melsungen, Germany). The injectability of the developed NEs was compared with those of reference emulsions, i.e., Intralipid^®^, Lipidem, and SMOFlipid. Water for injection was used as a control. A pressure threshold of 75 mmHg was established in this assay, and if this limit was exceeded during injection, an alarm was triggered. For the test, 30 mL samples were drawn into 50 mL syringes and administered at different infusion rates (25, 50, 75, 100, and 200 mL/h) as well as in bolus mode (800 mL/h) through needles of various gauges (23 G, 22 G, 21 G, 19 G, and 18 G). The occurrence of the alarm was monitored and recorded within the first minute of administration. All tests were conducted in triplicate.

#### 2.6.2. Hemolysis Activity

To evaluate the hemolysis activity of the prepared NEs, their effect on the human red blood cell was assessed. Briefly, 80 μL of NEs diluted in 2 mL of PBS were combined with 6 mL of red blood cell concentrate and incubated for 60 min at 37 °C under continuous shaking. After incubation, the samples were centrifuged at 1200 rpm for 10 min, and 2 mL of the supernatant was transferred to monovette tubes with EDTA. Pure red blood cell concentrate was also subjected to the same conditions as a control. Triton X-100 reagent and 0.9% NaCl solution were used for positive and negative controls, respectively. The hemolytic activity of the developed NEs was compared with those of the reference commercial intravenous lipid NEs Intralipid^®^, Lipidem, and SMOFlipid. The hemoglobin (HGB) concentration was determined using the XN-10 automated hematology analyzer (Sysmex America Inc., Lincolnshire, IL, USA), a dual-function FDA-approved analyzer, and the percentage of hemolysis was calculated according to the following equation:

(3) $$Hemolysis\;\left(\%\right)=\frac{C_{sample}-C_{negative}}{C_{positive\;}-C_{negative}}\times 100\%$$

where *C_negative_, C_positive_*, and *C_sample_* are the hemoglobin concentrations of the negative control, positive control, and sample, respectively.

#### 2.6.3. Compatibility Studies with PN

To evaluate the compatibility of the developed NEs with PN, the formulations were mixed with commercially available lipid-free PN admixtures: Omegaflex Special with electrolytes (OSE (+)) and Omegaflex Special without electrolytes (OSE (−)). Before the study, the PN admixtures were activated by combining the glucose and amino acid solutions from their respective chambers. The activated lipid-free PN admixtures were then mixed with the NEs in a 4:1 (*v*/*v*) ratio, reflecting the typical proportion between lipid emulsions and other components in commercial PN preparations. Physical stability tests, including visual inspection, MDD, PDI, and ZP measurements, were conducted immediately after mixing and after 24 h of storage. The test was carried out in triplicate.

### 2.7. The In Vitro Studies

The MTT assay was conducted according to the methodology for intravenous lipid NEs outlined in our previous study [22]. The THLE-2 (ATCC CRL-2706) cells were cultured in BEGM medium supplemented with the Bullet Kit (Lonza, Cologne, Germany) and 10% FBS (EURx, Gdansk, Poland), 5 ng/mL EGF, 70 ng/mL phosphoethanolamine (Sigma-Aldrich, St. Louis, MI, USA) in a humidified atmosphere with 5% CO_2_ at 37 °C. The medium was changed two to three times per week.

The MTT assay was conducted to evaluate the effect of the developed NEs on the viability of normal liver cells, compared with Intralipid^®^ and Lipid, using a standardized protocol. Briefly, THLE-2 cells (10^4^ cells/well) were seeded in a 96-well plate and preincubated for 24 h in a complete medium. Subsequently, the tested NEs, formulated as 20% oil-in-water emulsions, were diluted with the culture medium in appropriate proportions and added to the wells. Cells were treated with NEs at concentrations ranging from 0.1% to 7.4%, corresponding to dilution factors of approximately 2.7-fold to 270-fold. The cells were incubated for another 24 h. After incubation, the cells were washed with phosphate-buffered saline (PBS), and a fresh complete medium containing MTT salt (0.5 mg/mL) was added and incubated again for 4 h. In the final step, formazan crystals were dissolved in isopropanol containing HCl, and the absorbance was detected at 540 and 690 nm using the TECAN Infinite M200 plate reader. Statistical analysis of the results obtained was performed using the GraphPad InStat 3 version. 

### 2.8. Statistical Analysis

Results are expressed as mean values ± SD. The data were analyzed using Statistica 12 software (StatSoft Polska Sp. z o.o., Krakow, Poland). One-way analysis of variance (ANOVA) was used to determine the statistical significance among the samples. The a priori level of significance was *p* < 0.05.

## 3. Results and Discussion

### 3.1. Analysis of Fatty Acids Composition

The advancement of knowledge regarding the role of individual fatty acids in promoting health, maintaining optimal physiological functions, and influencing specific diseases is expected to drive the development of innovative, customized fat emulsions tailored to patients’ unique medical needs [23]. A similar trend is already evident in amino acid formulations used in PN, where specialized products are available for neonatal patients, adult patients, and those with liver failure or kidney dysfunction [24]. Applying a personalized approach to intravenous lipid emulsions could significantly enhance therapeutic outcomes, particularly in critically ill, oncological, or pediatric patients. In this study, we design an oil phase with a balanced lipid profile of fatty acids belonging to each of their families (saturated, monounsaturated, polyunsaturated fatty acids). A comparison of the lipid profiles of the selected oil phase and commercially available formulations, i.e., Intralipid^®^ (first-generation lipid emulsion), Lipidem, and SMOFlipid (third-generation lipid emulsions) is presented in Table 2. The composition of commercial lipid emulsions is given based on an analysis of the summary of product characteristics [25,26,27].

The proposed qualitative and quantitative compositions of oils allowed us to obtain an oil phase with 50% medium-chained fatty acids, a satisfactory amount of PUFA (in the range of the third-generation of lipid emulsion) with omega-6-to-omega-3 ratio equal to 2.8 and a reduced long-chain SFA content (3.8%) compared with commercial preparations: Intralipid (14.7%), Lipidem (6.5%), and SMOFlipid (8.9%).

Observational studies consistently show that dietary patterns with lower average intakes of SFA are associated with improved cardiovascular outcomes. Evidence supports the notion that replacing SFA with PUFA can reduce the risk of atherosclerotic cardiovascular disease. Consequently, current guidelines recommend limiting SFA intake to less than 10% of total daily energy for the general healthy population, with more stringent recommendations (e.g., 5–6% of total daily energy) for individuals with hypercholesterolemia [28]. On the other hand, increasing attention is being directed toward PUFA, particularly the optimal balance between omega-6 and omega-3 fatty acids that may prevent various pathological events [29]. Emphasis is also placed on the administration of biologically active omega-3 fatty acids, specifically eicosapentaenoic acid (EPA) and docosahexaenoic acid (DHA) in PN [30].

Given the above information, fatty acid profile analysis seems crucial to confirm that the profile allows for achieving the expected health-promoting or therapeutic effects associated with administering intravenous fat emulsions. In this study, as the first step of the proposed assessment protocol, such analysis was performed based on available literature data [3,14,15]. Although the analysis based on literature data was utilized and considered by us as acceptable, potential variations in the fatty acid content of oils—even from the same plant species—due to natural differences in fatty acid composition and the technological processes applied highlight the need for more precise methods in the case of the need for exact profile determination. In such a case, analytical techniques such as gas chromatography–mass spectrometry (GC–MS) can be employed, which was successfully utilized to determine the fatty acid profile in oil-in-water systems by Noureddine et al. [31] and Butt et al. [32]. Since the method used by those authors requires sample heating, which may lead to under-estimation of certain PUFAs, unstable at elevated temperatures, we suggest utilizing another transesterification method developed by Xu et al. [33] for the assessment of fatty acid profile in novel intravenous NEs. Xu et al. [33] developed an improved GC–MS method to determine fatty acid profiles in enteral nutrition or PN formulations, including intravenous lipid emulsions, which involves a one-step transesterification method that employs the addition of acetyl chloride in tubes kept in a dry ice bath, the transesterification at room temperature, and the data analysis using relative response factors.

### 3.2. Physicochemical Characterization of NEs

The second step of the proposed assessment protocol focuses on the physical characterization of NEs. Ensuring the safety and effectiveness of intravenous lipid emulsions necessitates meticulous attention to their physical properties. Critical parameters to evaluate include the absence of visible particles, the lipid emulsion’s droplet size, the oil-in-water system’s homogeneity, zeta potential, osmolarity, and pH levels. The Ph. Eur. [6] described in detail the methodology for the visual examination of parenteral preparations, including intravenous lipid emulsions. This simple method of examination is a critical quality control step to ensure the product’s safety and efficacy. The guidelines emphasize the absence of visible particulate matter when examined under suitable lighting conditions against a white and black background to detect any particles or abnormalities. Intravenous NE must be free from visible particulate contaminants or precipitates when inspected. Moreover, this methodology may also allow for the detection of the destabilization process, such as the appearance of oil droplets on the surface or inhomogeneities in the color intensity of the preparation. These phenomena indicate the onset of creaming, flocculation, or coalescence [34] and should not be observed in NEs for PN. The developed NEs, regardless of the type of co-surfactant used or its absence, showed no signs of destabilization or visual differences among themselves. All developed samples were milky-white homogeneous systems free from particulate contamination. The Ph. Eur. [35] provides specific guidelines for the assessment of subvisible particulate matter in parenteral preparations. These particles are not visible to the naked eye and require specialized instrumentation to quantify. This involves light obscuration particle count tests, where undiluted preparations are subject to the analysis of particles of the size greater than 10 µm and 25 µm, and the microscopic particle count test when the sample is filtered through 1.2 µm filter, and then the filter membrane is systematically examined. The microscopic evaluation is recommended particularly when the product’s characteristics, e.g., high viscosity or opacity, as in the case of intravenous NEs, make it unsuitable for light obscuration methods. For large-volume parenterals (≥100 mL), particles ≥10 µm cannot exceed the number of 25 particles per mL, and particles ≥25 µm cannot exceed the number of 3 particles per mL. Despite the methods for assessing visible and subvisual particles in intravenous preparations described in the Ph. Eur. [6,35], there is still a lack of tests and requirements specific to intravenous lipid emulsions, which can be found in the USP [2], which more precisely define the size of lipid emulsion particles. Therefore, in the case of assessing the physical properties of new intravenous NEs, we recommend using the methods described in the Ph. Eur. (visual examination and microscopic evaluation) and the USP (Method I and Method II) or using at least the methodology described in the USP. Methods I and II outlined in Chapter 729 of the USP specify that the MDD of tested samples, as determined using the DLS method, must not exceed 500 nm, additionally, the PFAT5 parameter, assessed using the LO method, must remain below 0.05%. The administration of NEs with values exceeding these limits may result in embolization of blood vessels and occlusion of the infusion catheter [2,36]. Method I using DLS is only reliable for samples with high homogeneity, i.e., those for which the PDI is less than 0.7 [37,38]. It is worth mentioning that Peng et al. reported better resistance to destabilization when the PDI value was less than 0.2 [20].

The physical characteristics of evaluated NEs differing in specific co-surfactants in comparison with commercial emulsions are presented in Table 3.

**Figure 2:**
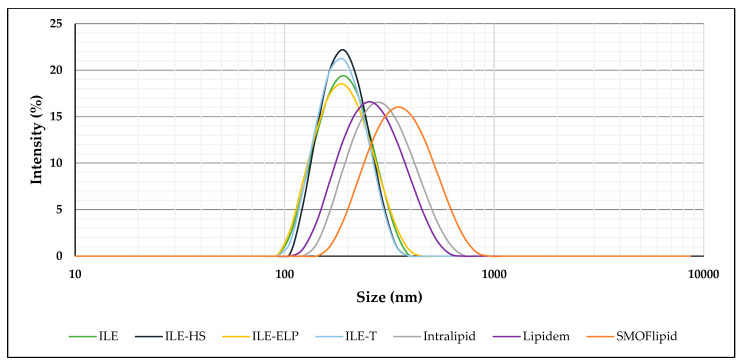
A correlogram of the particle size distribution of developed NEs and commercial formulations.

Despite adding various co-surfactants, obtained NEs were characterized by similar MDD values below 187 nm and low PDI values below 0.1. Compared with commercial emulsions, a significant difference can be seen in MDD, which was significantly higher in their case than in the developed NEs. Figure 2 shows a correlogram of the particle size distribution, confirming the homogeneity of developed and commercial NEs using the DLS method. The developed NEs exhibited a more uniform and narrower particle size distribution compared with commercial formulations.

The parameter differentiating the obtained NEs was PFAT5. In the case of ILE-HS, its value was 0.12%, which exceeded the adopted USP limit [2]. This indicates the importance of performing particle size measurements to characterize NEs for intravenous use using both methods. Despite the MDD being within the recommended standard, the PFAT5 value for ILE-HS disqualifies this NE from intravenous application. However, for the sake of experimental diversity during the proposed protocol validation, it was further investigated. It is also important to examine the underlying reasons for this phenomenon. Buszello et al. [39] observed that emulsions formulated with lecithin and Kolliphor^®^ HS15 demonstrated a tendency to destabilize following heat sterilization as the concentration of Kolliphor^®^ HS15 increased. This destabilization was attributed to exceeding the cloud point of aqueous Kolliphor^®^ HS15 solutions, which occurs at a temperature of approximately 75–80 °C. When this value is exceeded, the emulsifier dehydrates, leaves the interfacial layer, and forms independent micelles. Leakage of co-surfactants from the interfacial layer can initiate destabilization [39].

Another crucial parameter used to characterize NEs is ZP, which serves as an indicator of the stability of a colloidal system. ZP represents the surface charge generated by the emulsifier, which creates repulsive forces between particles. These repulsive forces counteract the attractive forces of van der Waals interactions, thereby enhancing the stability of the colloidal system and preventing particle aggregation. It is a value that depends on many factors, including pH changes or ions addition [3]. It has been assumed that NEs with absolute ZP values above 30 mV have good stability [40]. The surface charge distributed at the oil droplet boundary for ILE-T was −29.8 mV, and for ILE, ILE-HS, and ILE-ELP, it reached a similar value of about −32.5 mV. The ZP value at this level can be attributed to the combined effects of sodium oleate, used as a co-emulsifier, and egg yolk lecithin, which consists of a mixture of various phospholipids, including both neutral and anionic fractions. Together, sodium oleate and egg yolk lecithin form a cohesive interfacial layer around the emulsion droplets, ensuring electrical repulsion between oil droplets and contributing to the stability of the emulsion [41,42]. ZP analysis allows us to conclude that all developed NEs should show satisfactory stability.

pH and osmolarity (OSM) are critical parameters not only for the stability of NEs but also for evaluating their suitability for intravenous administration. Intravenous lipid NEs should ideally exhibit isohydricity; however, due to the blood’s high buffering capacity, slight deviations within the pH range of 6 to 9 are permissible. This range ensures compatibility with both peripheral and central vein administration, maintaining safety and efficacy [43]. When evaluating PN, tonicity is a crucial factor in preventing undesirable fluid shifts between intracellular and extracellular compartments. The osmotic agent most often added to intravenous lipid NEs is glycerol [44]. It is generally accepted that the maximum osmolarity for administration via peripheral veins is 900–1000 mOsm/L, while for central veins, it can safely reach up to 3000 mOsm/L. The osmolarity values of the individual components in a PN admixture should not exceed the recommended limits [45]. However, during the compounding process, the addition of components with high osmolarity, such as sodium chloride or potassium chloride, may elevate the overall osmolarity. Therefore, it is advisable to maintain the intravenous NE osmolarity within the range of physiological blood (275 to 295 mOsm/L) to ensure biocompatibility and safety.

### 3.3. Preliminary Stability Studies

Conducting preliminary stability studies is the next proposed step in the developed protocol for evaluating novel intravenous lipid NEs for PN. Stability studies are conducted to investigate the physical stability of the developed NEs, which is crucial for determining a new product’s expiration date. The principles for conducting drug stability studies are specified in the ICH Q1A–Q1F guidelines [46]. These guidelines regulate stability studies in terms of selection and number of batches, packaging materials, acceptance criteria, storage conditions, testing frequency, analytical methods, and labeling. This type of study is inherently tedious and time-consuming. Therefore, in this protocol, we propose conducting preliminary stability tests to evaluate the potential of novel NEs for further investigation. Among the available methods for predicting the stability of intravenous NEs, freeze–thaw testing, thermal cycling, autoclaving, and shaking tests are the most commonly used [21,47,48,49]. Interestingly, Han et al. [48] assessed the physical properties of two propofol emulsion formulations and found the most pronounced differences in their stability during shaking and freeze–thaw tests, despite their similar initial characteristics. On the other hand, Washington et al. [49], in their study comparing Intralipid and Ivelip, observed the most significant changes during autoclaving tests. Based on these findings, we recommend incorporating freeze–thaw and autoclaving tests (sterilization stability assessment) into the protocol to predict the stability of intravenous NEs.

#### 3.3.1. Freeze–Thaw Test

The freeze–thaw test, in particular, is critical for evaluating the robustness and stability of lipid emulsions under simulated environmental stress conditions. By subjecting emulsions to repeated cycles of freezing and thawing, this test measures their ability to maintain physicochemical stability and resist degradation or phase separation. Table 4 presents common conditions for the freeze–thaw test.

The proposed protocol in this methodology refines the standard freeze–thaw parameters. Unlike the most conventional approaches, which only involve freezing and thawing [47,48,49,50], our protocol, imitating Wang et al. [21], elevates the temperature to 40 °C after thawing. This additional step allows us to assess the impact of both decreased and increased temperatures, providing a more comprehensive evaluation of its stability. Additionally, following Wang et al. [21], the calculation of K_F_ was performed. The smaller the K_F_ value is, the better the freeze–thaw stability of the emulsion is. If the emulsion ruptures, the freeze–thaw test is incomplete, and K_F_ is recorded as +∞.

Analyzing the results, the most significant change was observed in the ILE formulation without additional co-surfactants. This formulation showed the largest increase in MDD of 4.1 ± 1.5 nm, along with a rise in ZP of 10.1 ± 1.2 mV and a slight increase in PFAT5 to 0.02 ± 0.01%. Despite these changes, the ILE parameters after the freeze–thaw test remained within the USP requirements for intravenous emulsions [2]. For ILE-HS, the freeze–thaw test reduced the PFAT5 value by 0.04 ± 0.02%; however, the final PFAT5 value still exceeded the established standards (Table 5). Conversely, ILE-ELP and ILE-T demonstrated excellent stability during the triple freeze–thaw cycle.

Analyzing K_F_, each value obtained is close to 0, confirming that the developed NEs remained stable during the freeze–thaw test (Table 6). It was observed that the K_F_ values for ILE-ELP and ILE-T were the lowest, supporting the previous conclusions regarding these two NEs. The K_F_ of developed NEs was in the range of those determined for commercial formulation (between 0.67 and 1.74).

#### 3.3.2. Sterilization Stability

The sterilization stability of intravenous NEs is crucial for maintaining their safety, therapeutic effectiveness, and structural integrity. It is essential that sterilization methods not only ensure the elimination of microbial contaminants but also preserve key properties such as particle size, zeta potential, and overall formulation stability. K_S_ is the parameter that, according to Wang et al. [21], may help determine the stability of NEs during thermal sterilization. The smaller the K_S_ value is (in other words, the smaller the change in the particle size before and after sterilization), the better the sterilization stability of the emulsion is.

**Figure 3:**
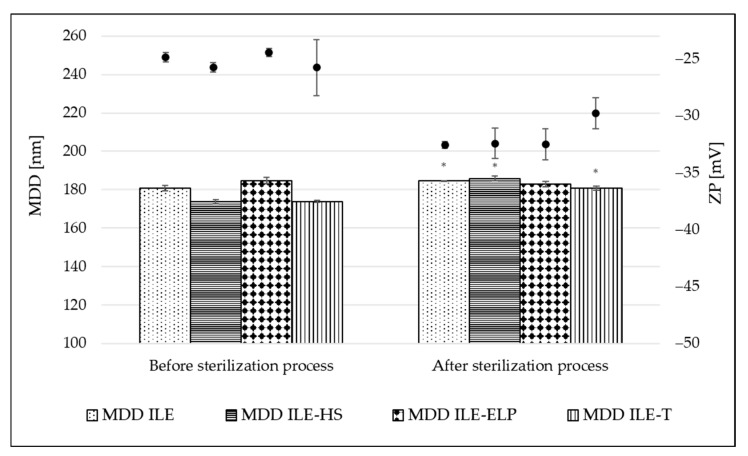
Results of MDD and ZP before and after thermal sterilization. *—results statistically significant; *p* = 0.05; MDD: mean droplet diameter; ZP—zeta potential. The left axis (100–260 nm range) refers to MDD, and the right axis (−50–−25 mV) and points above the bars refer to ZP.

The sterilization stability was evaluated by comparing the physiochemical parameters of NEs before and after the sterilization. Figure 3 shows the change in MDD and ZP parameters caused by the thermal sterilization. A small but significant increase in MDD values appeared in almost all tested NEs, ranging from 2.0% for ILE to 6.5% for ILE-HS of initial values. A non-significant change was observed only for ILE-ELP (Figure 3). The calculated K_S_ introduced by Wang et al. [21] also confirms the above observations (Table 6). The lowest value of 1.30 ± 1.16 was obtained for ILE-ELP, and a much higher value of 23.61 ± 1.65 for ILE-HS. The analysis of K_S_ allows significant conclusions to be drawn about the initial stability of NEs and, together with K_F_, allows a preliminary assessment of the stability of the obtained NEs based on MDD changes occurring during the respective processes. The sterilization process also significantly increased the absolute value of ZP in all formulations, with the most pronounced effect of 24.9% for ILE-ELP compared with the initial value. Two phenomena can explain the more negative surface charge of the micelles after thermal sterilization. One relates to the movement of egg yolk lecithin from the aqueous phase, where it exists as empty micelles, to the interface of oil droplets under heat. The second phenomenon relates to the release of free fatty acids from triglycerides due to lecithin degradation. As suggested by other researchers, the increase in the level of free fatty acids and the stabilized organization of the interfacial material lead to an increase in the stability of lecithin-based NE after thermal sterilization [41,51]. Additionally, a decrease in pH values by an average of 1 unit was observed for each formulation, which was also caused by the release of free fatty acids. Given that emulsion stability decreases significantly at pH below 5 units, it is worthwhile to plan to technologically increase the pH with a sodium hydroxide solution, if necessary, at the end of the NE preparation process so that the expected pH after sterilization is around 7 [3,51].

#### 3.3.3. Mid-Term Stability

**Figure 4:**
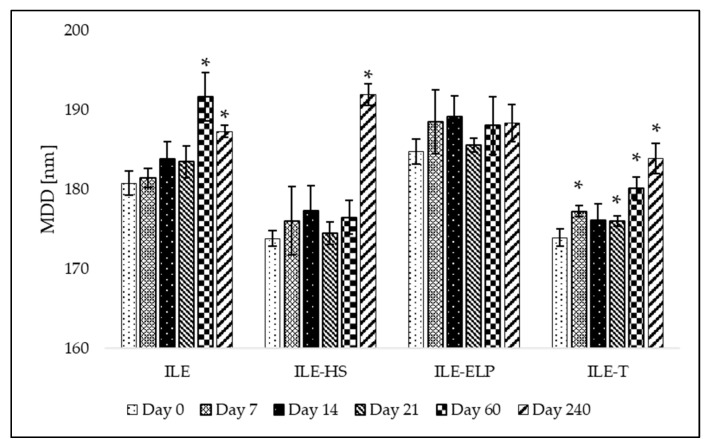
Mean droplet diameter (MDD) of developed NEs during mid-term stability tests. *—result statistically significant; *p* = 0.05.

**Figure 5:**
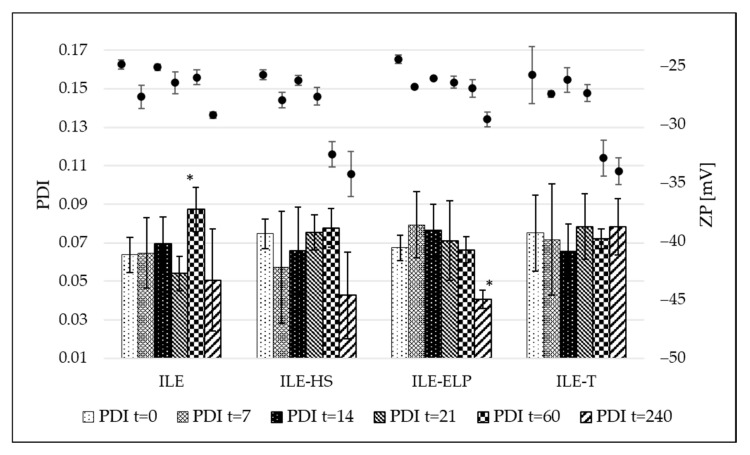
Results of polydispersity index (PDI) and zeta potential (ZP) of developed NEs during mid-term stability studies. *—result statistically significant; *p* = 0.05. The left axis 0.01–0.17 range refers to PDI, and the right axis −50–−25 mV and points above the bars refer to ZP.

The proposed preliminary stability test was validated through a mid-term stability assessment. The mid-term stability study involved the storage of developed NEs over 240 days at 4 ± 1 °C without exposure to light. The results presenting MDD changes and the fluctuation of PDI and ZP values at different time points during the test are shown in Figure 4 and Figure 5, respectively. An analysis of the mid-term study results revealed that changes in MDD were not significant for ILE-ELP throughout the study period. In contrast, significant changes in MDD were observed for ILE, ILE-HS, and ILE-T at specific time points (Figure 4). Nevertheless, none of the results exceeded the setup limit. The PDI value did not fluctuate significantly throughout the study in all samples except ILE on day 60 and ILE-ELP on day 240 (Figure 5).

The stability analysis of the developed NEs demonstrated that ILE-ELP was the most stable formulation throughout the study period (Figure 4 and Figure 5). No significant differences in MDD and ZP were observed during the entire storage period. Additionally, the decrease in PDI values after day 60 may indicate an improvement in NE homogeneity. These findings are consistent with the results of the freeze–thaw test and sterilization stability study, where a decrease in ZP values was noted, confirming the validity of the proposed methods for predicting NE stability. In the case of ILE-HS, a significant increase in MDD after 240 days (Figure 4) and a decrease in ZP were observed in the mid-term test. In contrast, results observed in the freeze–thaw test showed that PFAT5 for this formulation decreased. These may indicate an increase in the stability of the formulation and the transformation of large particles into smaller ones that are within the detection range of the Zetasizer apparatus, which could have contributed to the increase in MDD. However, despite a slight decrease in the PFAT5 value of ILE-HS, it still did not meet *USP* requirements for intravenous administration (Table 5) [2].

Summing up, the mid-term stability test results underscored the reliability, cost-effectiveness, and simplicity of the freeze–thaw and sterilization stability tests as preliminary stability assessment tools for lipid NEs. These methods provide critical insights into formulation robustness and offer a practical framework for researchers to optimize formulations, ensuring enhanced long-term stability.

### 3.4. Verifying the Suitability for Intravenous Administration as a Component of PN

#### 3.4.1. Injectability Assay

The next step in the proposed protocol for assessing NEs involves verifying their suitability for intravenous administration as a component of PN. This involved the evaluation of the injectability of NEs, the lack of hemolysis effect, and the compatibility of NEs with the remaining components of PN.

One of the key factors in ensuring the safety of intravenous drug delivery is the evaluation of the rheological properties of NEs [52]. While neither the USP nor the Ph. Eur. provides specific requirements for measuring injectability, several established methods can be employed to assess the feasibility of NE administration. Injectability can be determined through various approaches, such as an arbitrary score system, utilizing a texture analyzer, estimating the force required to expel the formulation from a syringe using a force-testing apparatus, or employing an infusion syringe pump equipped with an occlusion pressure alarm [22,53,54]. This last method involved the comparison of the pressure exerted during the infusion of the developed formulation with that of a reference formulation. This methodology was employed in the proposed assessment protocol.

The results for the developed NEs were compared with those obtained for commercial intravenous NEs widely used in the medical field (Intralipid^®^, Lipidem, and SMOFlipid), and the water for injection was used as a neutral comparator. This study utilized needles of various sizes to trigger a pressure alarm set at a threshold of 75 mmHg.

The results of the injectability test are shown in Table 7.

The type of surfactant used and the oil phase composition were shown to affect the emulsion’s ability to be injected. Comparing the reference samples, Intralipid^®^ allows infusion through 22 and 21 G needles, but only at a maximum infusion rate of 25 and 50 mL/min, respectively. Moreover, using the thickest 18 G needle, the maximum possible flow rate is 75 mL/min. In the case of Lipidem, it is possible to infuse through a 22 G needle at a maximum rate of 25 mL/min, but once changed to a 21 G needle, continuous infusion can be conducted at all rates applied. SMOFlipid flow through the 19 G needle does not cause alarm at all tested flow rates. However, changing to a 22 or 21 G needle allows infusion at a maximum 50 mL/min rate. Analyzing the results obtained for commercial intravenous emulsions and considering that the maximum infusion rate for such emulsions in adults is approximately 50 mL/h and the most often used catheter size is 20 G, we established the following acceptance criteria for the injectability testing protocol: to qualify as injectable, the tested NE formulation must not trigger the pressure alarm at the set threshold when infused through at least a 20 G needle at a rate of 50 mL/h.

This study allowed us to observe that Intralipid^®^, which bases its formulation on soybean oil, has significantly worse injectability than Lipidem and SMOFlipid, which contains both soybean oil, MCT, and Ω-3 acid triglycerides or fish oil in their formulations (Table 2). This shows that the composition of the oil phase is essential in terms of the rheological properties of NE and, thus, the patient’s sensation during drug administration. The higher the percentage of MCT, the lower the viscosity of the NE [55]. This is confirmed by the fact that all developed NEs showed a better injectability profile than Intralipid^®^. The results collected for the developed NEs showed the worst performance of ILE-HS, in which infusion can be carried out only using a needle with a minimum diameter of 19 G. The results of this test align with the physicochemical parameters identified for ILE-HS in the previous assessment. In the case of ILE-ELP, the results were better than those for SMOFlipid and similar to those for Lipidem. Better injectability compared with Lipidem was demonstrated only by ILE-T, in which a 22 G needle allowed infusion at a maximum rate of 100 mL/min and a 23 G needle at a rate of 25 mL/min. Regarding the acceptance criteria, only ILE-ELP and ILE-T demonstrated satisfactory injectability, as they could be infused at a rate of 50 mL/h using a 21 G needle. ILE based solely on egg yolk lecithin caused pump alarm at lower rates than Lipidem or SMOFlipid. Using lecithin as an emulsifier significantly increases the viscosity of NEs and, thus, the observed problem with their injectability [56]. The results show that the presence of a suitable co-emulsifier is crucial to reducing the viscosity of the formulation and facilitating the drug administration.

#### 3.4.2. Hemolysis Test

Another important aspect highlighted in this assessment protocol is the performance of a hemolysis assay. This test is critical for evaluating the biocompatibility of the developed NEs and ensuring their safety for intravenous administration. It provides valuable insights into the potential of the formulations to cause damage to red blood cells, which is a key parameter for clinical acceptability. According to the literature and the applied methodology, hemolysis thresholds of 5% [57] or 10% [58] were defined. Therefore, we established an acceptance limit of 10%. To assess the hemolytic activity of novel NEs with different co-emulgators, red blood cells were incubated with tested formulations, and commercially available intravenous NEs (Intralipid^®^, Lipidem, and SMOFlipid) were used as references. The hemolytic effect was tested against positive and negative controls, and the results are shown in Table 8. All developed NEs showed hemolytic activity within the most restricted criteria (5%) as well as in the range of values measured for Intralipid^®^, Lipidem, and SMOFlipid equal to 2.16%, 3.60%, and 3.60%, respectively. Such results suggest that the studied NEs do not cause the breakdown of red blood cells and can be safely administered intravenously in future in vivo studies.

#### 3.4.3. Compatibility Test

**Figure 6:**
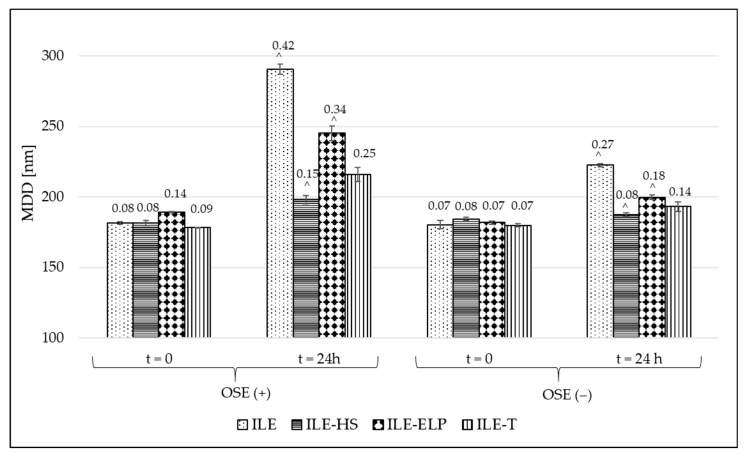
Results of compatibility tests. MDD: mean droplet diameter; OSE (+): Omegaflex^®^ Special with electrolytes; OSE (−): Omegaflex^®^ Special without electrolytes; ^: the appearance of the second fraction of lipid droplets above 4000 nm. The polydispersity index values are presented above each bar.

The last proposed test to confirm the feasibility of the intravenous administration of the developed NE as a macronutrient in PN is compatibility testing with commercial multi-chamber bags (MCBs). The proposed test is a novel approach and, to the best of our knowledge, has never been described before in the literature. The compounding of a PN consists of combining intravenous NEs with the remaining components of the aqueous phase of PN, i.e., a solution of amino acids, glucose, and electrolytes. Confirmation of the compatibility of the formulated novel NEs for PN with the remaining components of the admixture is an essential element of the development of a new product. Such a complete product must be stable for at least 24 h, i.e., from the moment of preparation until the end of the mixture supply to the patient. In this protocol, 3-in-1 PNs provided in MCBs were utilized. The idea of 3-in-1 preparations in MCBs is to enable a prolonged shelf life by the separation of the aqueous phase containing electrolytes, i.e., amino acids and glucose solutions, from lipid emulsion. Such mixtures are available in several variants, including electrolyte-free formulations, which allow the selection of the appropriate composition, corresponding to the patient’s needs. In the proposed test, both electrolytes containing PN and electrolyte-free formulation were used to define the effect of electrolytes on the compatibility of developed NEs with the remaining PN components. Briefly, the substitution of commercial NEs presented in MCBs with the tested formulations was performed, and the physicochemical parameters, such as MDD, PDI, and ZP, were determined. In the compatibility verification, for assessing the globules found in the large-diameter tail of the resulting PN admixture instead of PFAT5 evaluation, the absence of a second fraction of lipid droplets greater than 4000 nm using the DLS method was used [59,60]. The tested NEs were combined with the aqueous phase of OSE (+) and OSE (−). Figure 6 illustrates the physical parameters determined during the compatibility testing. Measurements were taken at two time points: immediately after combining the components and after 24 h of storage at room temperature, protected from light. These time points were selected based on the minimum storage requirements of the complete PN admixture, aligned with the maximum duration of PN infusion, which is 24 h [61].

When analyzing the compatibility results for developed NEs combined with PNs containing and not containing electrolytes, it was noted that none of the developed NEs showed signs of destabilization immediately after preparation. However, after 24 h of storage, all tested NEs exhibited significant changes in MDD (*p* < 0.05), and in all samples except ILE-HS and ILE-T combined with OSE (-), the DLS analysis revealed larger lipid droplet size fractions above 4000 nm. The lack of larger droplets in ILE-HS and ILE-T was also confirmed by minor variations in PDI values compared with other NEs. Despite the favorable compatibility results, ILE-HS was rejected from further investigations due to its failure to meet the acceptance criteria for PFAT5 during the physiochemical characterization. Consequently, only ILE-T appears to have the greatest potential for use in PN admixtures; however, the appearance of large droplets in electrolytes-containing PN requires further investigations to establish the maximum amounts of electrolytes ensuring the stability of the completed formulation.

### 3.5. In Vitro Assay

Finally, evaluating the novel intravenous lipid NE for PN requires a thorough assessment of its safety profile before proceeding to in vivo models. In the literature, various cell lines are employed for assessing intravenous formulations, including the following:

- MRC-5: A diploid cell culture line composed of fibroblasts, originally derived from the lung tissue of a 14-week-old aborted Caucasian male fetus [55];
- EVC304: A spontaneously transformed cell line originating from human umbilical vein endothelial cells [62];
- L-929: A fibroblast-like cell line derived from the subcutaneous connective tissue of a 100-day-old male C3H/An mouse [63];
- Vero: Cell lines isolated from kidney epithelial cells of an African green monkey [64];
- HEK-293: A cell line derived from the kidney of a human embryo [65];
- HSC-T6: An immortalized hepatic stellate cell line developed from primary stellate cells of male Sprague-Dawley retired breeder rats, transformed with the SV40 large T-antigen [66];
- THLE-2: Immortalized human adult liver epithelial cells modified with recombinant simian virus 40 large T-antigen [22];
- BRL 3A: A fibroblast-like cell isolated from the liver of a rat [67].

**Figure 7:**
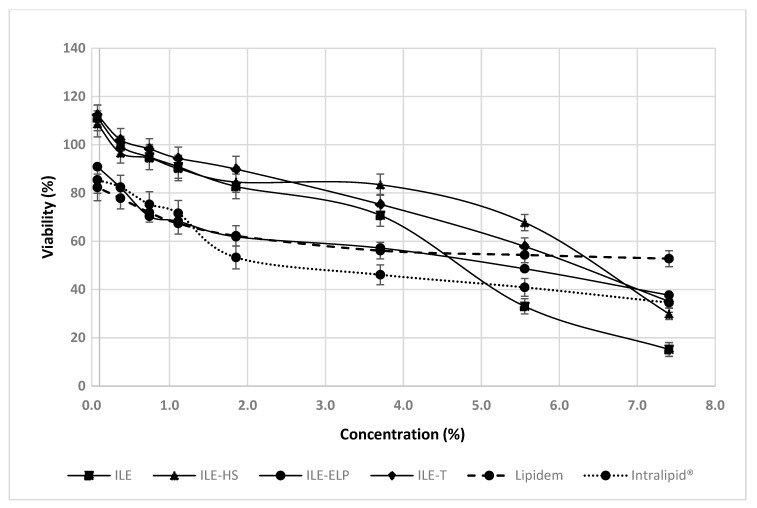
The effect of novel intravenous NEs on the viability of THLE-2 cells. Data (mean ± SEM) from three separate experiments are presented.

Each of these cell lines offers unique advantages for evaluating the safety and efficacy of intravenous lipid formulations. However, considering the hepatotoxic potential of intravenous lipid NEs used in PN, which is a contributing factor in the pathogenesis of intestinal failure-associated liver disease [4,5,68], we recommend assessing the hepatotoxic potential of novel NEs as a gold standard. Performing an MTT test on the HSC-T6, THLE-2, BRL 3A, or other normal liver cell lines provides invaluable insights before transitioning to in vivo experiments, thereby helping to prevent liver damage in laboratory animals. In this protocol, the assessment of hepatotoxic potential was determined using the THLE-2 cell line. According to Pastor-Clerigues et al. [69], a 1:100 emulsion dilution (equivalent to a 0.2% emulsion concentration) yields a fatty acid concentration of 200 mg/dL, comparable to plasma levels seen in humans after a 1.2 g/kg dose of parenteral lipid emulsion. This dilution represents the lowest concentrations examined in the proposed protocol. However, to evaluate the potential toxic effects of novel NEs comprehensively, we performed the MTT assay across a broad range of concentrations, including less diluted samples, consistent with approaches used by other investigators studying the effects of parenteral NEs in vitro [67,69]. Our findings demonstrate that all the tested NEs, except for ILE-ELP, exhibited better cell viability at low concentrations (up to approximately 4%) compared with commercial NEs (Intralipid^®^ and Lipidem), which served as references (Figure 7). On the other hand, the most pronounced concentration-dependent negative effect on cell viability between the lowest and highest concentrations was observed for ILE. For this reason, ILE-HS and ILE-T appear to be the most promising candidates for further research. However, considering all the results obtained in this protocol and the failure of ILE-HS to meet the pharmacopeial requirements for PFAT5, only ILE-T can be approved for subsequent animal studies.

## 4. Conclusions

This study aimed to develop and validate a comprehensive in vitro assessment protocol tailored to evaluate novel intravenous lipid NEs for PN before releasing them for in vivo testing. By employing the proposed five-step evaluation, comprising (1) fatty acid composition analysis; (2) physicochemical characterization; (3) assessment of injectability, compatibility, and biocompatibility; (4) preliminary stability testing; and (5) MTT assay, the safety and potential of novel formulations for further development can be comprehensively assessed. This protocol provides a standardized framework for evaluating novel intravenous lipid NEs, ensuring their minimal physicochemical performance and stability, biocompatibility, and suitability for PN. Employing such an approach adheres to rigorous bioethical standards, minimizing the risk of exposing animals to formulations with unsatisfactory properties.

## Figures and Tables

**Table 1:** Composition of developed NEs.

Composition	ILE	ILE-HS	ILE-ELP	ILE-T
	(% *w*/*w*)			
MCT	10.0	10.0	10.0	10.0
Sunflower oil	3.2	3.2	3.2	3.2
Canola oil	3.2	3.2	3.2	3.2
Fish oil	2.0	2.0	2.0	2.0
Hemp seed oil	1.6	1.6	1.6	1.6
α-tocopherol	0.02	0.02	0.02	0.02
Lipoid^®^ E 80	1.2	1.2	1.2	1.2
Kolliphor^®^ HS15	-	0.25	-	-
Kolliphor^®^ ELP	-	-	0.25	-
Tween^®^ 80	-	-	-	0.25
Glycerol	2.25	2.25	2.25	2.25
Sodium oleate	0.05	0.05	0.05	0.05
Water	ad 100.0	ad 100.0	ad 100.0	ad 100.0

**Table 2:** The oil and fatty acid compositions of the design NEs and commercially available lipid emulsions.

Components	Designed NEs	Intralipid^®^	Lipidem	SMOFlipid
Oil composition (%)				
Soybean oil	-	100	40	30
Olive oil	-	-	-	25
Canola oil	16	-	-	-
Sunflower oil	16	-	-	-
Hemp seed oil	8	-	-	-
MCT	50	-	50	30
Fish oil	10	-	-	15
Omega-3 fatty acid triglycerides	-	-	10	-
Fatty acids composition (%)				
Caprylic ^1^	26.4	0	27.8	16.5
Capric ^1^	26.4	0	27.8	16.5
Palmitic ^1^	2.5	10.8	4.8	6.9
Stearic ^1^	1.3	3.9	1.7	2.0
Oleic ^2^	14.9	24.0	10.6	27.9
α-linolenic ^3^	3.4	7.8	3.5	2.7
EPA +DHA ^3^	3.5	0	7.2	5.4
Linoleic ^4^	19.4	52.3	23.2	19.8

DHA: docosahexaenoic acid; EPA: eicosapentaenoic acid. ^1^ SFA—saturated fatty acids. ^2^ MUFA—monounsaturated fatty acids. ^3^ PUFA Ω-3—polyunsaturated fatty acids Ω-3. ^4^ PUFA Ω-6—polyunsaturated fatty acids Ω-6. The percentage of each fatty acid listed in the table is approximate and was calculated based on literature data [3,14,15].

**Table 3:** Characterization of developed NEs after sterilization (results expressed as mean value ± standard deviation, n = 3).

Parameter	ILE	ILE-HS	ILE-ELP	ILE-T	Intralipid^®^	Lipidem	SMOFlipid
MDD (nm)	184.5 ± 0.2	185.9 ± 1.1	182.9 ± 1.5	180.7 ± 0.6	273.7 ± 4.4	249.6 ± 5.0	335.4 ± 4.9
PDI	0.06 ± 0.01	0.07 ± 0.02	0.05 ± 0.00	0.06 ± 0.02	0.11 ± 0.00	0.10 ± 0.00	0.08 ± 0.02
PFAT5 (%)	0.01 ± 0.005	0.12 ± 0.009	0.02 ± 0.007	0.01 ± 0.003	0.01± 0.00	<0.01	<0.01
ZP (mV)	−32.6 ± 0.3	−32.4 ± 1.3	−32.5 ± 0.5	−29.8 ± 0.6	−38.5 ± 0.4	−37.3 ± 0.6	−43.9 ± 1.1
pH	6.8 ± 0.02	7.1 ± 0.01	7.1 ± 0.01	6.9 ± 0.07	8.0 *	6.0–8.5 *	8.0 *
OSM (mOsm/kg)	368 ± 2.8	374 ± 5.6	378 ± 3.5	378 ± 7.7	350 *	410 *	380 *

*—data from summary of product characteristics [25,26,27].

**Table 4:** Literature review of the methodology of the freeze–thaw test.

Test Type	Temperature Range	Cycle Number	Reference
Freeze–thaw	Freeze at −20 °C for 48 h, followed by thaw at 40 °C for 48 h	3 cycles	Wang et al., 2021 [21]
Freeze–thaw	Freeze at −18 °C for 16 h, followed by 8 h of thawing at room temperature	1–7 cycles	Damitz et al., 2015 [47]
Freeze–thaw	Freeze at −20 °C for 8 h, followed by thawing at room temperature	1 cycle	Han et al., 2001 [48]
Freeze–thaw	Freeze in liquid nitrogen for at least 15 min, followed by thawing in a water bath near 20 °C	1 cycle	Komatsu et al., 1996 [50]
Freeze–thaw	Freeze at −20 °C for 15 h, followed by thawing at room temperature	1 cycle	Washington et al., 1993 [49]

**Table 5:** Change in MDD, PDI, ZP, and PFAT5 parameters after freeze–thaw tests.

Sample	ΔMDD (nm)	ΔPDI	ΔZP (mV)	ΔPFAT5 (%)
	Observed Change ± SD			
ILE	↑4.1 ± 1.5	↑0.02 ± 0.02	↓10.1 ± 1.2	↑0.02 ± 0.01
ILE-HS	↑0.7 ± 2.3	↑0.03 ± 0.01	↓6.7 ± 1.4	↓0.04 ± 0.02
ILE-ELP	↓0.6 ± 2.2	↑0.03 ± 0.01	↓4.1 ± 0.8	↓0.01 ± 0.01
ILE-T	↓2.6 ± 1.8	↓0.01 ± 0.02	↑5.5 ± 1.0	↓0.01 ± 0.00
Intralipid^®^	↓2.6 ± 3.8	↑0.01 ± 0.00	↓8.9 ± 1.6	<0.01
Lipidem	↓2.6 ± 3.4	↑0.00 ± 0.00	↓3.5 ± 0.5	<0.01
SMOFlipid	↑1.8 ± 5.8	↑0.06 ± 0.02	↑2.1 ± 0.8	<0.01

SD—mean standard deviation from before and after freeze–thaw test, **↑**—increase, **↓**—decrease.

**Table 6:** Results of freeze–thaw stability constant (K_F_) and sterilization stability constant (K_S_) for developed NEs.

Sample	ILE	ILE-HS	ILE-ELP	ILE-T	Intralipid^®^	Lipidem	SMOFlipid
K_F_	1.21 ± 1.00	1.18 ± 0.87	0.87 ± 0.34	0.92 ± 0.59	1.00 ± 0.04	0.67 ± 0.21	1.74 ± 0.53
K_S_	3.16 ± 2.30	23.61 ± 1.65	1.30 ± 1.16	8.51 ± 3.41	n.d.	n.d.	n.d.

n.d.—not determined.

**Table 7:** Results of the injectability test.

Needle Size [G]	Infusion Rate [mL/h]	Water for Injection	Intralipid^®^	Lipidem	SMOFlipid	ILE	ILE-HS	ILE-ELP	ILE-T
23	25	+	–	–	–	–	–	–	+
	50	+	–	–	–	–	–	–	–
	75	–	–	–	–	–	–	–	–
	100	–	–	–	–	–	–	–	–
	200	–	–	–	–	–	–	–	–
	800	–	–	–	–	–	–	–	–
22	25	+	+	+	+	–	–	+	+
	50	+	–	–	+	–	–	–	+
	75	+	–	–	–	–	–	–	+
	100	+	–	–	–	–	–	–	+
	200	+	–	–	–	–	–	–	–
	800	+	–	–	–	–	–	–	–
21	25	+	+	+	+	+	–	+	+
	50	+	+	+	+	–	–	+	+
	75	+	–	+	–	–	–	+	+
	100	+	–	+	–	–	–	+	+
	200	+	–	+	–	–	–	+	+
	800	+	–	+	–	–	–	–	+
19	25	+	+	+	+	+	+	+	+
	50	+	+	+	+	+	+	+	+
	75	+	–	+	+	+	+	+	+
	100	+	–	+	+	+	+	+	+
	200	+	–	+	+	+	+	+	+
	800	+	–	+	+	+	+	+	+
18	25	+	+	+	+	+	+	+	+
	50	+	+	+	+	+	+	+	+
	75	+	+	+	+	+	+	+	+
	100	+	–	+	+	+	+	+	+
	200	+	–	+	+	+	+	+	+
	800	+	–	+	+	+	+	+	+

“–”: infusion impossible to perform (occlusion pressure alarm triggered); “+”: continuous infusion possible to perform.

**Table 8:** Results of the hemolysis test performed on NEs.

Sample	Hemolysis (%)
ILE	2.16
ILE-HS	2.88
ILE-ELP	3.60
ILE-T	3.60
Intralipid^®^	2.16
Lipidem	3.60
SMOFlipid	3.60

## Data Availability

The datasets used and/or analyzed in the current study are available from the corresponding author upon reasonable request.

## Abbreviations

The following abbreviations are used in this manuscript:

3R	Replacement, Reduction, and Refinement
DHA	docosahexaenoic acid
DLS	dynamic light scattering
EPA	eicosapentaenoic acid
GC–MS	gas chromatography–mass spectrometry
HGB	hemoglobin
IFALD	intestinal failure-associated liver disease
IV	intravenous
K_F_	freeze–thaw stability constant
K_S_	sterilization stability constant
MCB	multi-chamber bag
MCT	medium chain triglycerides
MDD	mean droplet diameter
MUFA	monounsaturated fatty acids
NE	nanoemulsion
OSE (+)	Omegaflex^®^ Special with electrolytes
OSE (−)	Omegaflex^®^ Special without electrolytes
OSM	osmolality
PC	phosphatidylcholine
PDI	polydispersity index
Ph. Eur.	European Pharmacopeia
PN	parenteral nutrition
PNALD	parenteral nutrition-associated liver disease
PFAT5	percentage of fat residing in globules larger than 5 µm
PUFA	polyunsaturated fatty acids
SD	standard deviation
SFA	saturated fatty acids
USP	United States Pharmacopeia
ZP	zeta potential
